# Magnitude and its associated factors of teenage pregnancy among antenatal care attendees in Bahir Dar city administration health institutions, northwest, Ethiopia

**DOI:** 10.1186/s12884-022-05130-y

**Published:** 2022-10-29

**Authors:** Fentahun Yenealem Beyene, Azimeraw Arega Tesfu, Kihinetu Gelaye Wudineh, Toyiba Hiyaru Wassie

**Affiliations:** Department of Midwifery, College of Medicine and Health Sciences, Bahir Dar University, Bahir Dar, Ethiopia

**Keywords:** Teenage, Pregnancy, Bahir Dar city

## Abstract

**Background:**

Worldwide teenage pregnancies develop many devastating complications, both the mother and the neonate like developing anemia, nutritional deficiency, pregnancy induced hypertension, preterm baby, inadequate weight gains and obstructed labor, fistula and sepsis. Reproductive health concerns of adolescents the main emphasis area which increasing international attention in recent years. Therefore, we intended to assess the magnitude and its associated factors of teenage pregnancy in Bahir Dar city administration health institutions, northwest, Ethiopia, 2017.

**Methods:**

A health institution based a cross-sectional study was conducted among pregnant mothers from February 20-March 27, 2017 in Bahir Dar city administration. Five hundred forty-nine participants were selected by face to face interview and medical card review by using systematic random sampling technique every four intervals for each health institution. Bivariate and multivariate data analysis was performed using Statistical Package for the Social Sciences (SPSS) Windows version 21 and level of significance of association was determined at *P*- value < 0.05.

**Result:**

The study identified 12.2%with (95%CI (9.5, 14.9)) of pregnant women were teenagers. Multivariable logistic regression analysis showed that: [(AOR (95% CI)) rural residency 3.21(1.234, 9.345), age at first marriage < 18 years 9(7.823, 17.571) and not using contraception prior to this pregnancy 5.22(3.243, 11.675)] were significantly associated with teenage pregnancy.

**Conclusion:**

The magnitude of teenage pregnancy was comparable to the 2016 Ethiopian demographic health survey finding. Rural residency, age at first marriage and not using of contraception prior to the current pregnant were significantly associated with teenage pregnancy. As per the findings, awareness creation to the rural population, advocating utilization of contraception, avoid early marriage and put the mindset the effect of teenage pregnancy for those are needed.

## Introduction

World Health Organization (WHO) defines the age group 10–19, 13–19 and 15–24 years of age as adolescents, teenagers and youth, respectively [1, 2]. Globally, up to 1.2 billion of the world's population make in the age group of 15–24 years. Majority of them live in Sub-Saharan Africa and vulnerable to teenage pregnancies and HIV infection and unintended pregnancy [3–5].

In each year, around 21 million girls aged 15 to 19 years and 2 million girls aged less than 15 years become pregnant in developing regions [6, 7]. Worldwide every year, 16 million women and girls faced to pregnancies within the age of 15 to 19 years [8, 9].

According to Save the Children report, annually, 13 million children are born to women under age 20 worldwide of these more than 90% in developing countries. Complications of pregnancy and childbirth are the leading cause of mortality among women between the ages of 15 and 19 [10].

Reproductive health concerns of adolescents the main emphasis area which increasing international attention in recent years. Even though early pregnancies are higher in the world; more pronounced in Sub-Saharan African (SSA) countries, like Kenya which results forced to drop out of school or get married at an early age, inability to meet basic and personal material needs makes teenage girls susceptible to pre-marital sex and predispose them to unwanted pregnancies [11].

According to the Ethiopian Demographic Health Survey (EDHS 2016) data showed that 13 percent of women age 15–19 in Ethiopia have begun childbearing: 10 percent have had alive birth and 2 percent were pregnant with their first child at the time of interview [12].

Worldwide teenage pregnancies develop many devastating complications in different aspects, both the mother and the neonate like health related consequences (developing anemia, nutritional deficiency, pregnancy induced hypertension, preterm baby, inadequate weight gain and obstructed labor, fistula, develops sepsis) and behavioral, economic and social related consequences (smoke, drink, or take drugs during pregnancy, they may not avail antenatal services or come late to the health, some may also seek unsafe abortions, teenage mothers dropping out of school, remaining unmarried or unemployed and living in poverty) [1, 2, 13–15]. The reviewed literature showed that, residency, maternal education, partner education, lack of parent to adolescent communication on sexual and reproductive health (SRH) issues, marital status, and inadequate opportunity in community level for positive youth development, illiteracy, poverty and limited employment opportunities and age at mirage and contraceptive utilization were the main determinants of teenage pregnancy [16–22]. There is no study in the study area, despite numerous studies being conducted throughout Africa, at the national level, including in the Amhara region. Therefore, we intended to assess the magnitude and its associated factors of teenage pregnancy.

## Methods

### Study area

A study was carried out in Bahir Dar city administration; a regional city of Amhara region. The city is located 565 km far from Addis Ababa, the capital city of Ethiopia. In the city there are ten governmental health centers, one regional referral hospital, and one district hospital owned by the government. In the city there are 17 urban administrative kebeles and 4 special towns with a projected total population of 297,794 in the year 2016 [23].

### Study design and period

A health institution based a cross-sectional study was conducted from February 20 to March 27, 2017.

### Source and study population

All pregnant women who attended ANC at all government health institutions in Bahir Dar city administrations.

### Inclusion criteria

All pregnant mothers who are attending ANC selected governmental health institutions in Bahir Dar city administrations.

### Sample size determination

The sample size was determined by using a single population proportion formula which took the following assumptions in to consideration: the proportion of teenage pregnancy 20.4% [24], 5% level of significance (α = 0.05) and 5% margin of error (ω = 0.05). The final sample size was adjusted by adding 10% non-response rate and considering design effect 2 thus turned out to be 549.

### Sampling procedure and technique

Seven governmental health institutions were selected out of 12 governmental health institutions through random lottery method. Information about the client flow to each health institution was obtained from Amhara regional health bureau [25]. The average client flow of the selected health institutions was taken from registry book of the selected health institutions. The data were almost similar, which ranges from 16 to 23 clients per day and this number was multiplied with monthly working days which was 22 days. The total sample size was proportionally allocated for 7 health institution depending on the client flow in each health institution. For each health institution the first participant selected randomly; then the subsequent participants were selected by systematic random sampling technique every four intervals for each health institution.

### Data collection tools and procedures

Data were collected through face to face exit interviews and medical card review using a structured and pre-tested questionnaire. The tool first prepared in English then translated to Amharic and back to English by language expert to maintain the consistency of the instrument. Seven diploma holder nurses conducted the face to face interviews and two BSc degree midwives supervised the data collection process.

### Data quality assurance

The collected data were checked for the completeness, accuracy, clarity and consistency after conducting pre-test. A pre-test was conducted on 40 pregnant mothers in one of the health centers out of the study area called Merawi Health Center and the instrument was amended accordingly. Any error, ambiguity or incompleteness identified was corrected immediately. The data collectors were trained for one day about the contents of the questionnaire, methods of data collection and aim of the study. The data collection process was supervised by the supervisor and the investigator throughout the data collection period.

### Data processing, analysis and interpretation

Data were coded, cleaned and entered by Epi. Info version 7 and analyzed using computer database software and transported to the SPSS version 23 statistical software. Descriptive statistics like frequencies and percentages were used to present the categorical independent variables and mean/standard deviation was used to describe a continuous variable. Frequency tables and graphs were used to present descriptive results. For this study, bivariate logistic regression model was fitted as a primary method of analysis. Odds ratios (OR) were computed with the 95% confidence interval (CI) to see the ANC time of initiation in relation to the considered associated factors in this research. Independent factors, with a *P*-value < 0.2 obtained in the bivariate logistic regression were entered into the multiple logistic regression models. Consequently, the most important associated factors were identified using the multivariate logistic regression analysis. Then an adjusted odd ratio (AOR) with 95% confidence interval were calculated for the significant predictive variables, and statistical significance was accepted at (*P* < 0.05).

## Results

### Socio-demographic characteristics of the respondents

The study included a total 549 pregnant mothers with response rate of 100%.Among the total respondents four hundred twenty (76.5%) urban in residency. Orthodox Christianity was the dominant religion which is 91.8%. Almost all of the respondents were Amhara by ethnicity. Five hundred thirty-two (96.9%) respondents were married (Table 1).

**Table 1:** Percentage distribution of the study population by socio demographic characteristics; Bahir Dar City Administration, Ethiopia, February 20- March 27, 2017(*n* = 549)

Variables	Categories	Frequency	Percentage
Residences	Urban	420	76.5
	Rural	129	23.5
Ethnic group	Amhara	541	98.5
	Others^a^	8	1.5
Religion	Orthodox	504	91.8
	Muslim	40	7.3
	Others^b^	5	.9
Marital status	Married	532	96.9
	Single	13	2.4
	Others^c^	4	0.7
Women Educational level	Un able to write and read	172	31.3
	Primary school(1–8)	170	31.0
	Secondary and above	207	37.7
Women Occupational level	Employed	112	20.4
	Employed Self	35	6.4
	House wife	362	65.9
	Daily laborer	64	7.3
Partner Educational level	Un able to read and write	130	24.3
	Primary school(1–8)	127	23.7
	Secondary and above	279	52
Average family monthly income	< = 400	42	7.7
	400–1000	53	9.7
	> 1000	454	82.7

^a^Oromo, Tigre

^b^Catholic, Protestant

^c^Divorced, Widowed

### Reproductive characteristics of the respondents

Majority of the respondent’s age at marriage were greater than or equal to eighteen years which was 54.3% and more than two-third of them their age at first pregnancy were above eighteen years (78.9%). Three hundred fifty-two (64.1%) respondents were multiparous. Around four hundred fourteen (75.4%) respondents were their pregnancy planned (Table 2).

**Table 2:** Percentage distribution of the study population by reproductive characteristics; Bahir Dar City Administration, Ethiopia, February 20-March 27, 2017

Variables	Categories	Frequency	Percentage
Age at marriage	< 18	251	45.7
	> = 18	298	54.3
Age at first pregnancy	< = 18	116	21.1
	> 18	433	78.9
Parity of respondent	Nulliparous	197	35.9
	> = 1	352	64.1
Was the pregnancy planned?	Yes	414	75.4
	No	135	24.6
Have you ever heard aboutFP?	Yes	518	94.4
	No	31	5.6
Have you ever used at least one method of FP?	Yes	489	89.1
	No	60	10.9
Type of method you have ever used recently?	Pills	23	4.6
	Depo Provera	280	56.3
	Implant	93	18.7
	IUCD	75	15.1
	Emergency	2	.4
	Male condom	23	4.6
**Reason for not used FP**	Never had sexual intercourse	8	13.3
	Forced sex	7	11.7
	Want to get pregnant	30	50
	Lack of family consent	15	25

*IUCD* Intra Uterine Contraceptive Device, *FP* Family Planning

### Current teenage pregnancy

**Fig. 1:**
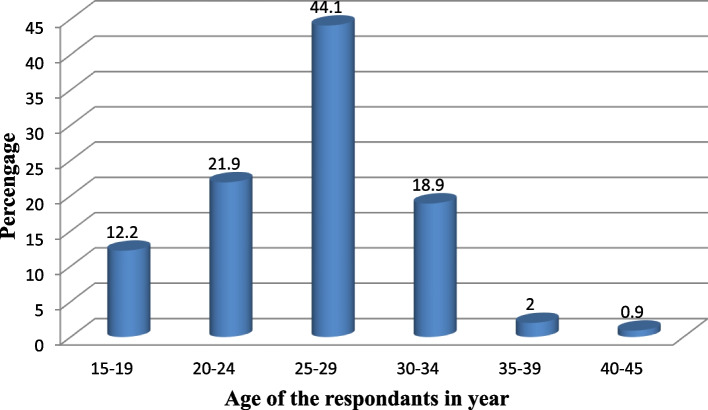
Percentage distribution of the study population by age category; Bahir Dar City Administration, Ethiopia, February 20- March 27, 2017(*n* = 549)

This study identified that, 12.2% with (95%CI (9.5, 14.9)) participants were their current pregnancy within the age of 15–19 years (Fig. 1).

### Factors associated with teenage pregnancy

Bivariate analysis was done to assess any association between independent variables and teenage pregnancies. In bivariate analysis: residence, women education, age at first marriage, knowing about family planning methods, utilization of contraception and parity were considered statistically significant with teenage pregnancy. Multivariable logistic regression analysis showed that women with living in rural area were four times more likely to be teenage pregnancy compared to urban residency (AOR [95% CI] = 3.21(1.234,9.345. Likewise, mothers whose age at first marriage was below eighteen years were more likely to be teenage pregnancy (AOR [95% CI] = 9(7.823, 17.571) and Women that not utilizes contraception prior to this pregnancy were more likely to have teenage pregnancy (AOR [95% CI] = 5.22(3.243, 11.675) (Table 3).

**Table 3:** Logistic Regression analysis of socio-demographic and reproductive factors for teenage pregnancy; Bahir Dar City Administration, Ethiopia, February20-March 27, 2017

Variables	Categories	Age at current Pregnancy in year			COR (95%CI	AOR (95% CI)
		** < = 19**		** > 19**		
Residence	Rural	39		90	6.07(3.547,10,377) `1	3.21(1.234,9.345)* 1
	Urban	28		392		
Women educational status	Not read & write	35	137		.07(.027, .222) .10(.034, .291) 1	
	Primary(1–8)	28	170			
	Secondary& above	4	203			
Have you ever Used FP?	No	28	32		1 10.09(5.521,18.462)	1 5.22(3.243,11.675 ) **
	Yes	39	450			
Have you ever heard about FP?	Yes	47	471		1 .055(.025,.121)	
	No	20	11			
Party of respondent	Nulli parous	64	133		.018(.006,.058) 1	
	> = 1	3	349			
Age at first marriage in year	< = 18	63	188		24.63(18.421,35.243)1	9(7.823, 17.571)* 1
	> 18	4	294			

^*^pvalue < 0.05

^**^pvalue < 0.001

## Discussion

This study showed that around 12.2% of pregnant women were teenagers. The proportion of teenage pregnancy was comparable to the 2016 EDHS finding which is 13% [12]. The proportion of teenage pregnancy was low in this study compared with the finding from Assosa general hospital which was 20.4% [24], this difference might be due to the fact that the time gap of the study; as the study near to this century their knowledge towards the bad outcome of teenage pregnancy and early marriage and attitudes towards contraception usage updated and also due to different study population. The finding of teenage pregnancy was low in this study compared with the finding from Kampala, Uganda which is four in every ten teenagers attending Naguru teenage Centre are pregnant [26]. This is might be due to the fact that the difference in the study population; the previous study was conducted on teenagers that may significantly increases the prevalence of teenage pregnancy. And also the finding of this study was lower than Kibuku Town, Eastern Uganda which was35.8% [27]. This might be due to sociocultural and norm difference. Another study in Northeast, Ethiopia showed that the prevalence of teenage pregnancy was higher than the current study which was 28.6% [28]. This might be due to difference in socio demographic factors and the study population and study sitting. On the contrary the finding in this study was higher than compared with study from Vietnam (4%), Arba Minch Town 7.7% [17, 29] respectively. This might be due to sociocultural and study population difference. In this study, socio-demographic, obstetric and information factors related to teenage pregnancy. Teenage pregnancy was significantly related to their residency. Women in rural residents were more likely to be teenage pregnancy than those who was urban. This finding agrees with the studies done in south Asia and northeast Ethiopia [28, 30] respectively; this might be evidenced by rural women are far by information, media and may have family pressure to early marriage than urban women. The analysis showed that respondents who were not using a contraceptive were more likely to being teenage pregnancy than those had experience of contraceptive utilization; these finding is similar with the study conducted in [24, 31–34] this might be the fact that contraception service is the ideal service for discussing timing of first pregnancy and in other perspective if they got the service there is no pregnancy until they want. The study showed that women with age at first marriage <  = 18 years old were more likely become teenage pregnancy than the counter part with. This explanation might be due to the fact that as the women marriage at the early age the tendency of being pregnant at teenage age is obviously high. In other perspective at early age marriage, the probability of having or access to media, information and contraceptive utilization is obviously low.

Some variables might be missed in the study such as: Age at first intercourse, type of sex you faced and family status and which might affect the current coverage of teenage pregnancy. In addition, community based studies that can address widely.

### Limitation of the study

Since the data collectors were nurses, there may be social desirable responses bias for some of the variables.

## Conclusion

In conclusion, the magnitude of teenage pregnancy was comparable to the 2016 EDHS finding.

Being rural residence Not uses contraception and age at first marriage <  = 18 years were significantly associated with teenage pregnancy. As policy level; we recommend that the regional health bureau create awareness for rural women regarding to the timing of first pregnancy and drawback of teenage pregnancy as well as promoting family planning utilization for delaying pregnancy and avoiding early marriage.

## Data Availability

The datasets used and/or analyzed during the current study are available from the corresponding author on reasonable request.

## Abbreviations

HIV: Human Immune Virus
STI: Sexual Transmitted Infection
EDHS: Ethiopian Demography Health Survey
WHO: World Health Organization
